# Infectivity studies of *Leishmania (Leishmania) infantum chagasi* isolated from non-ulcerated cutaneous leishmaniasis

**DOI:** 10.1590/S1678-9946202567021

**Published:** 2025-04-04

**Authors:** Gabriela Venicia Araujo Flores, Carmen Maria Sandoval Pacheco, Thaise Yumie Tomokane, Wilfredo Humberto Sosa Ochoa, Fernando Tobias Silveira, Concepción Zúniga, Carlos Eduardo Pereira Corbett, Rodrigo Pedro Pinto Soares, Luiz Felipe Domingues Passero, Marcia Dalastra Laurenti

**Affiliations:** 1 Universidade de São Paulo, Faculdade de Medicina, Laboratório de Patologia de Moléstias Infecciosas, São Paulo, São Paulo, Brazil; 2 Universidad Nacional Autónoma de Honduras, Instituto de Investigaciones en Microbiologia, Tegucigalpa, Honduras; 3 Instituto Evandro Chagas, Belém, Pará, Brazil; 4 Universidade Federal do Pará, Belém, Pará, Brazil; 5 Hospital Escuela, Departamento de Vigilancia de la Salud, Tegucigalpa, Honduras; 6 Fundação Oswaldo Cruz, Instituto René Rachou, Belo Horizonte, Minas Gerais, Brazil; 7 Universidade Estadual Paulista, Campus do Litoral, Instituto de Biociências, Departamento de Ciências Biológicas e Ambientais, São Vicente, São Paulo, Brazil; 8 Universidade Estadual Paulista, Instituto de Estudos Avançados do Mar, São Vicente, São Paulo, Brazil

**Keywords:** Non-ulcerated cutaneous leishmaniasis, Leishmania (Leishmania) infantum chagasi, Infectivity, Experimental infection, Hamster

## Abstract

In Honduras, *Leishmania* (*Leishmania*) *infantum chagasi*, the etiological agent of visceral leishmaniasis (VL), is responsible for non-ulcerated cutaneous leishmaniasis (NUCL). We characterized NUCL and VL Honduran strains to understand intraspecies infectivity. Based on *in*-*vitro* assays, we aimed to elucidate certain host-parasite interactions in VL and NUCL isolates through a hamster model. To assess the capacity of these strains to infect peritoneal macrophages, we exposed them to promastigotes from NUCL and VL patients at varying temperatures and time intervals (32, 34, and 36 °C; 24 and 48 h) and infection-index (II) was determined. No significant differences were observed over time for dermotropic strains; however, a higher II was noted at lower temperatures (32 and 34 °C). Interestingly, only the VL strain exhibited a higher II at elevated temperatures (34 and 36 °C) at 48 h. Low levels of oxygen and nitrogen-derived metabolites were detected in both NUCL and VL strains. For *in-vivo* assays, hamsters were infected subcutaneously (SC) and intraperitoneally (IP) with 10^7^-promastigotes from NUCL and VL patients. After 90 days of infection, parasite-load and histopathological changes were assessed from spleen samples. Regardless of the administration route, no substantial differences were observed in the histopathological features between NUCL and VL strains. In conclusion, lower temperatures may favor parasite infection for NUCL strains, mirroring conditions found in the skin. This contrasts with the VL strain, which demonstrated a superior II at higher temperatures, a condition normally found in the viscera. Our data also indicate that *M. auratus* is susceptible to Honduran *L.* (*L.*) *infantum chagasi* strains, circumventing the skin barrier by IP or SC injection.

## INTRODUCTION

In leishmaniasis, the initial stages of host-parasite interaction are critical to the infection's outcome. Key components of the host defense that are vital for eradicating *Leishmania* include elements of both the innate and adaptive immune systems^1^. Once in the skin, *Leishmania* seeks to invade, establish, and maintain infection via direct interactions between structures expressed on the parasite's surface with their corresponding receptors present on the membrane of host cells, mainly macrophages^2^. This capacity to evade the immune response is a significant factor in the pathology of leishmaniasis. It contributes to the challenges in eliminating the infection, particularly in its severe forms, such as visceral leishmaniasis (VL). In the case of *L*. (*L*.) *infantum chagasi* in Honduras, the clinical manifestation can either be benign non-ulcerated cutaneous leishmaniasis (NUCL) or fatal VL, depending on the host's immunological state. Notably, these clinical forms occur in the same geographical region but never simultaneously in the same patient, nor does one form precede the other. The capacity of *L.* (*L.*) *infantum chagasi* to visceralize or remain in the skin may be determined by the host's immune response, parasite genetics, or vector's saliva^3,4^. Early studies hypothesized that NUCL could result from continuous exposure to bites in endemic areas, modulating the host's immune responses in a protective way^3^. Moreover, an analysis based on the DNA polymerase alpha subunit gene revealed that *L. (L.) infantum chagasi* from Honduras possesses genetic characteristics indicative of greater ancestry than *L. (L.) infantum chagasi* from Brazil and Europe, suggesting that the ancient host-parasite relationship may also play a role in defining the atypical cutaneous leishmaniasis caused by a viscerotropic strain in Honduras^5^. Although significant interest has been directed towards understanding vascularization in *Leishmania* parasites, many mechanisms underlying this process in *L.* (*L.*) *infantum chagasi* remain unknown, especially in isolates of the same species. Hamsters inoculated with *L. (L.) infantum chagasi* subcutaneously demonstrate vascularization of the parasite within hours of infection, as evidenced by the presence of dividing amastigotes in the cytoplasm of macrophages in the draining lymph node 24 h after infection^6^. However, the persistence of a chronic inflammatory response at the cutaneous inoculation site does not prevent the vascularization of the infection, characterized by histopathological changes in the spleen and liver, along with intense tissue parasitism^7^. Furthermore, studies involving hamsters infected with dermotropic and viscerotropic strains of *Leishmania* indicate that, regardless of the route of inoculation, the parasites exhibit a specific tissue tropism. This phenomenon may be temperature-related, with viscerotropic parasites migrating or remaining in the spleen and liver, while dermotropic parasites localize in the skin^8^.

Despite recent efforts to understand NUCL/VL in Central America, information regarding parasite biology in animal models remains scarce^9^.

As part of a broader study on Honduran *L.*(*L.*)*infantum chagasi* strains, we comparatively evaluated their *in vitro* and *in vivo* biological behavior using peritoneal macrophages and hamsters (*Mesocricetus auratus*) as an experimental model. These strains constitute a valuable biological material isolated from a patient cohort over the past decade and may be instrumental in understanding the immunopathological mechanisms within the subgenus *Leishmania*.

## MATERIALS AND METHODS

### Ethics statement

Parasite isolation from human subjects in Amapala and Piraera, Honduras, adhered to procedures approved by the Research Ethics Committee of the Master of Infectious and Zoonotic Diseases at the National Autonomous University of Honduras (Nº 03-2014). Animal procedures followed protocols approved by the Committee on Ethics in the Use of Animals and the National Council for the Control of Animal Experimentation of the Medical School of Sao Paulo University (Nº 172/14). All experiments were conducted in strict accordance with the recommendations of the Guide for the Care and Use of Laboratory Animals of the Brazilian National Council of Animal Experimentation.

### Parasite isolation and molecular characterization

Parasites from four NUCL patients (skin scraping): MHOM/HND/2017/AMA-65 (AMA-65), MHOM/HND/2017/AMA-73 (AMA-73), MHOM/HND/2018/AMA-161 (AMA-161), and MHOM/HND/2018/AMA-614 (AMA-614) from Amapala; and one VL patient (peripheral blood): MHOM/HND/2020/LV-3 (LV-3) from Piraera, Honduras, were isolated. Culturing was performed according to Flores *et al.* ^9^. These regions are endemic for both clinical forms, NUCL and VL, in southern Honduras, with a prevalence of 73.6% for *L. (L.) infantum chagasi* infection^10^. The samples for parasite isolation were collected before treatment and after diagnosis; all patients received treatment following the protocol of the Ministry of Health of Honduras^11^.

Promastigote DNA was extracted using the *QIAamp DNA Mini Kit* (^©^QIAGEN, Germany), and PCR-RFLP reactions were conducted for the heat-shock protein 70 KDa gene (*hsp*70)^12^. Controls included the World Health Organization (WHO) reference strains: *L.*(*L.*)*infantum chagasi* (MHOM/BR/72/BH46) (BH-46), *L.* (*L.*) *infantum* (MHOM/BR/1974/PP75) (PP75), *L.* (*V.*) *panamensis* (MHOM/PA/2021/C1218) (C1218), *L.* (*V.*) *braziliensis* (MHOM/BR/1995/M15280) (M15280), and *L.* (*L.*) *amazonensis* (MHOM/BR/1973/M2269) (M2269). Following *hsp*70 amplification, fragments were digested with the restriction enzyme HaeIII (Promega^©^, USA) before *Leishmania* species identification in 3.5% agarose gels.

### *In vitro* infection

Male golden hamsters (*M. auratus*) (45–60 days old, 90–150g) were obtained from the Animal Facility of the Medical School of Sao Paulo University. Resident peritoneal macrophages were harvested following the injection of 10 mL of sterile saline buffered with 0.01 M phosphate pH 7.2 (PBS) into the peritoneum. The cell suspension was collected, placed in 15 mL tubes on ice, and washed three times with sterile PBS (180 ×g, 10 min, 4 °C). Attached cells (10^5^/coverslip) were cultured in Roswell Park Memorial Institute (RPMI) 1640 (Gibco, USA) supplemented with 10% fetal bovine serum (FBS), 1% of 100 µg/mL streptomycin, 100 IU/mL penicillin, 1 mM sodium pyruvate, and 1% v/v non-essential amino acid solution (Thermo Fisher, USA). As previously standardized and used in various studies^13,14^, macrophages were infected with 10^6^ parasites (10 parasites:1 macrophage) isolated from chronically infected hamsters, and plates were incubated for 24 and 48 h at 32 °C, 34 °C, and 36 °C with 5% CO^2^ in a humid atmosphere. At each time point, coverslips were washed with sterile PBS, fixed with methanol, and stained with Giemsa. The number of parasites per macrophage and intracellular amastigotes was quantified to determine the infection index (II), as reported by Yamamoto *et al*.^15^. Parasite growth curves were established, and the logarithmic and stationary growth phases were identified. In all experiments, parasites from the 5^th^ day of culture (stationary growth phase), at the 2^nd^ or 3^rd^ passages, were employed.

### Nitric oxide and hydrogen peroxide production

Culture supernatants were collected at each time point (24 and 48 h). Nitric oxide production was detected using the Griess Reagent System following the manufacturer's instructions at 550 nm (Promega^©^, USA). Hydrogen peroxide production was determined using the Amplex Red Hydrogen Peroxide/Peroxidase Assay following the manufacturer's instructions (Invitrogen^TM^, USA). Fluorescence was measured using a spectrophotometer, with excitation at 525 nm and emission at 590 nm (GloMax^®^-Multi+ Detection System, Promega^©^, USA).

### *In vivo* infection

Golden hamsters were infected with two NUCL strains (AMA-65 and AMA-73) and one VL strain (LV-3). Stationary phase parasites (10^7^/0.1 mL) were inoculated subcutaneously (SC) into the right hind footpad (n = 5) and via the intraperitoneal (IP) route (n = 5). At 90 days post-infection, animals were anesthetized and euthanized. A spleen fragment was collected for histopathological analysis using paraffin sections stained with hematoxylin-eosin (HE) and parasite load determination via limiting dilution assay as previously reported^16^.

Briefly, the spleen fragment was weighed, macerated, and diluted in Schneider medium (Sigma-Aldrich, USA) supplemented with 10% FBS and 1% of 100 µg/mL streptomycin and 100 IU/mL penicillin (Gibco, EUA). After pre-dilution, 150 µL of the suspension was added in four replicates to a 96-well sterile microplate (Jet-Biofil). Then, 11 serial dilutions (1:3) of the suspension were performed, and the microplates were incubated at 25 °C for 10 days. After this period, promastigote forms were observed in each well. The parasite load was expressed as the number of parasites per milligram of homogenized organ.

### Statistical analysis

Data analysis was conducted using the software GraphPad Prism (version 10, GraphPad Software, Boston, MA, USA), with a p-value < 0.05 considered statistically significant. Results are expressed as mean ± standard error. The student's t-test and One-way ANOVA were employed to analyze the infection index (II), nitric oxide determination, hydrogen peroxide production, and parasite load.

## RESULTS

### Molecular typing

**Figure 1:**
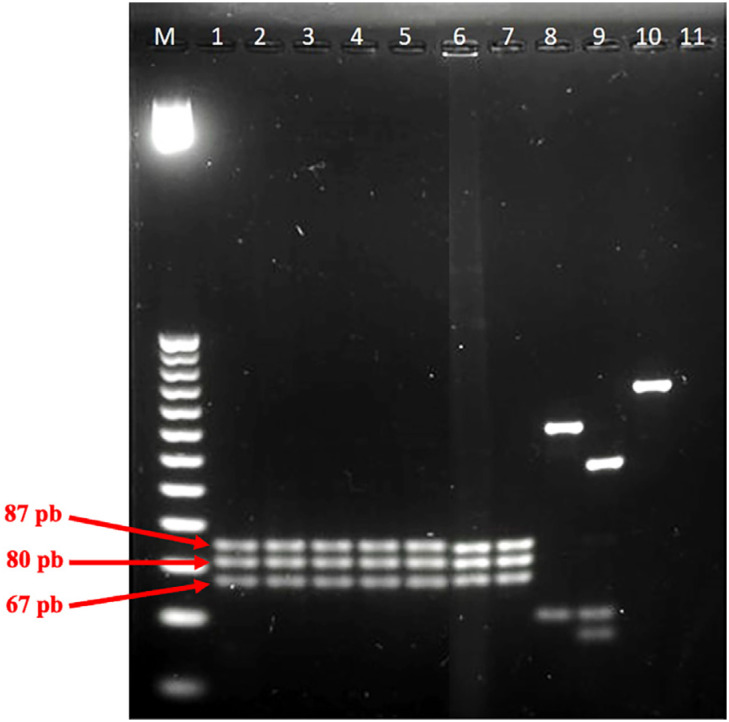
Molecular typing of Honduran *Leishmania* strains (dermotropic/viscerotropic) using PCR-RFLP of *Hsp*70 after *Hae*III digestion. Legend: M: 25 bp-Molecular weight; lanes 1–4: NUCL strains (AMA-65, AMA-73, AMA-161. and AMA-614); lane 5: VL strain (LV-3); Lanes 6–10, Controls: *L.* (*L.*) *infantum chagasi* (BH-46, lane 6 and PP75, lane 7), *L.* (*V.*) *panamensis* (C1218, lane 8), *L.* (*V.*) *braziliensis* (M15280, lane 9), and *L.* (*L.*) *amazonensis* (M2269, lane 10); and lane 11: negative control.

After *hsp*70 amplification followed by digestion with the restriction enzyme *Hae*III, fragments of 67 bp, 80 bp, and 87 bp were expected for *L. (L.) infantum chagasi* typing. All five Honduran strains (dermotropic/viscerotropic) were confirmed as *L.* (*L.*) *infantum chagasi* (Figure 1, lanes 1–5) since their profiles were identical to those of the control strains (PP75 and BH-46, lanes 6–7) and distinct from other species used as controls (lanes 8–10).

Clinical and epidemiological aspects: 100% (5/5) of patients with NUCL and VL from endemic areas in Honduras were female. The mean age of patients with NUCL was 38 years (range: 4–69 years), whereas the patients with VL was 1 year. Among patients with NUCL, three had a single lesion (75%) and one had multiple lesions (25%). The time of evolution of the lesions was ≤1 year in 75% (3/4) of cases and >1 year in 25% (1/4) of cases. The 1-year-old female patient with VL presented with a 1-month history of fever, hepatosplenomegaly, thrombocytopenia, anemia, leukopenia, and lower-extremity edema. Laboratory diagnosis was conducted using the rK39 immunochromatographic test (InBios International, USA). Table 1 summarizes these data.

**Table 1:** Clinical and epidemiological characteristics of the samples used in this study.

Case number	State	Municipality	Age (years)	Sex	Number of lesion(s)	Time of evolution of skin lesion (months)	Strain
**1**	Valle	Amapala	46	F	1	8	MHOM/HN/2017/AMA-65
**2**	Valle	Amapala	4	F	1	4	MHOM/HN/2017/AMA-73
**3**	Valle	Amapala	69	F	2	36	MHOM/HN/2018/AMA-161
**4**	Valle	Amapala	34	F	1	12	MHOM/HN/2018/AMA-614
**5**	Lempira	Piraera	1	F	-	1	MHOM/HN/2020/LV-3

F = Female.

### *In vitro* infection

To evaluate the infective potential of NUCL and VL Honduran strains at different temperatures, infection indices (II) in hamster macrophages were analyzed.

**Figure 2:**
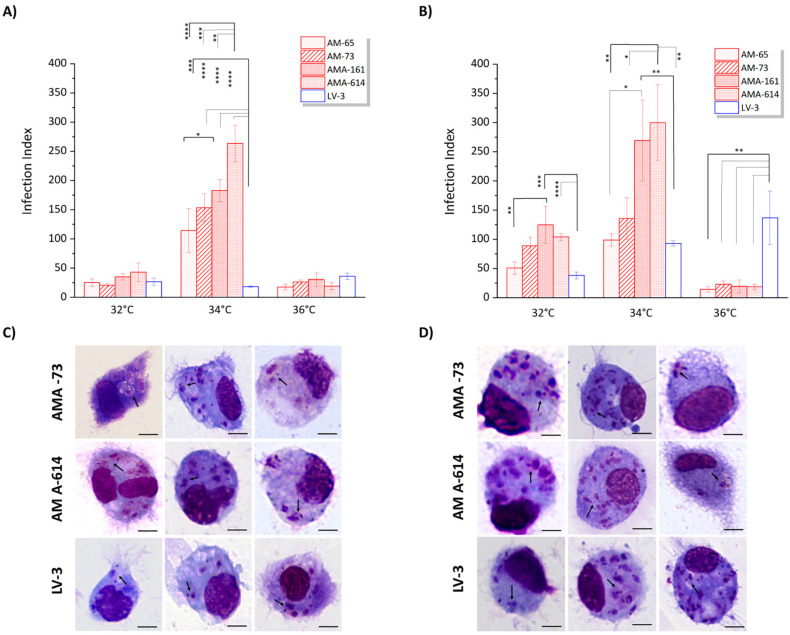
Infection index (II) in hamster macrophages infected with Honduran *L.* (*L.*) *infantum chagasi* strains at different temperatures: (A and C) 24 h post-infection; (B and D) 48 h post-infection; AMA-65, AMA-73, AMA-161, and AMA-614 represent dermotropic strains; and LV-3 represents the viscerotropic strain. *: *p* < 0.05; **: *p* < 0.01; ***: *p*<0.001; ****: *p* < 0.0001.

After 24 h of incubation and when comparing different temperatures (Figure 2A), significant differences were observesd only at 34 °C, in which a higher II was noted for dermotropic strains compared to the viscerotropic strain isolated from Honduran patients. At 32 °C and 36 °C, no differences were observed. After 48 h of infection (Figure 2B), more pronounced differences in II were found at 32 °C, 34 °C, and 36 °C. A higher II was recorded for dermotropic strains, particularly for AMA-73, AMA-161, and AMA-614, compared to the viscerotropic strain at 32 °C (*p* < 0.05). A similar result was noted at 34 °C, in which dermotropic strains exhibited a higher II than the viscerotropic Honduran strain (LV-3). Conversely, at 36 °C, the viscerotropic strain (LV-3) demonstrated a higher II than all dermotropic strains of *L. (L.) infantum chagasi* (*p*< 0.001).

Consistent with these observations, all strains were capable of infecting hamster macrophages at both time points, as evidenced by representative images of two dermotropic strains (AMA-73 and AMA-614) and one viscerotropic strain (LV-3) (Figures 2C and 2D).

### Nitric oxide (NO) and hydrogen peroxide production

**Figure 3:**
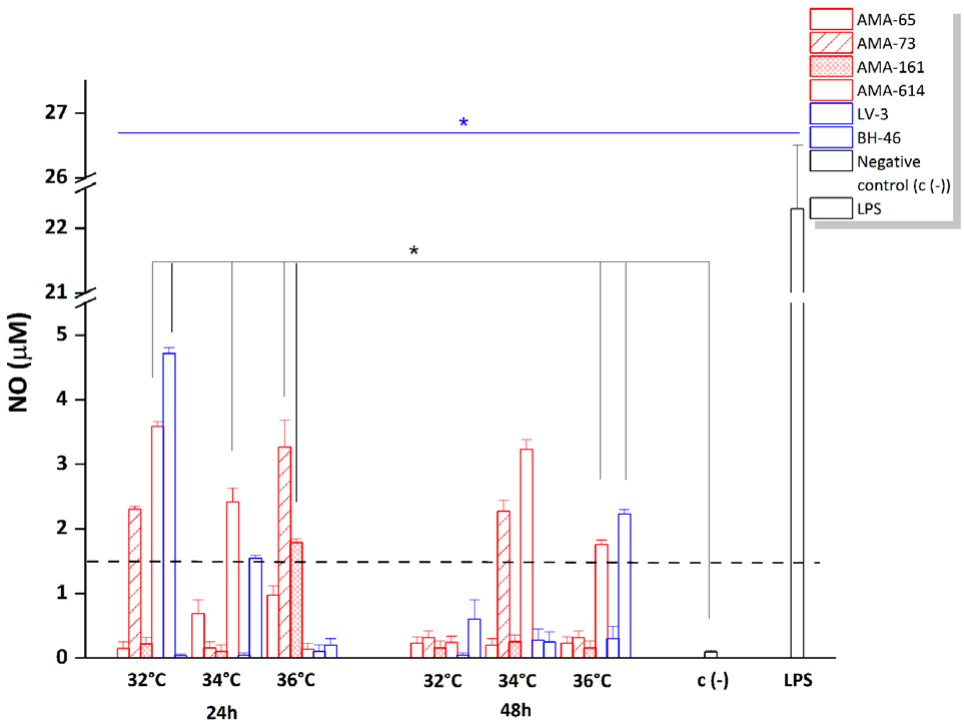
Nitric oxide production (µM) by hamster macrophages infected with Honduran *L.* (*L.*) *infantum chagasi* strains at different temperatures. Legend: at 24 h and 48 h post-infection; AMA-65, AMA-73, AMA-161, and AMA-614 represent dermotropic strains; and LV-3 represents the viscerotropic strain. The black line with asterisk indicates *p* < 0.05 between negative control and the samples; and the blue line with asterisk indicates *p* < 0.05 between LPS and the samples. The dashed line indicates the cut-off detection point.

The same supernatants of infected hamster macrophages were analyzed to evaluate the production of reactive oxygen intermediates. In most conditions tested, hydrogen peroxide production was minimal (below the reaction cut-off). Only AMA-65 infection at 48 h of incubation produced detectable hydrogen peroxide in the supernatant at 34 °C (0.16 µM; data not shown). In contrast to hydrogen peroxide, nitric oxide (NO) production was detected and varied among the different strains, incubation temperatures, and times of infection; however, it did not correlate with the II (Figure 3). After 24 h, NO production by macrophages was evident for some dermotropic strains, including AMA-73, AMA-161, and AMA-614, as well as the viscerotropic strain LV-3 (Figure 3). After 48 h, NO levels were detected at 32 °C, with higher levels persisting for AMA-73 and AMA-614 at 34 °C. These levels decreased at 36 °C, except for AMA-614 (Figure 3).

### *In vivo* infection

#### Histopathological changes and parasite load

**Figure 4:**
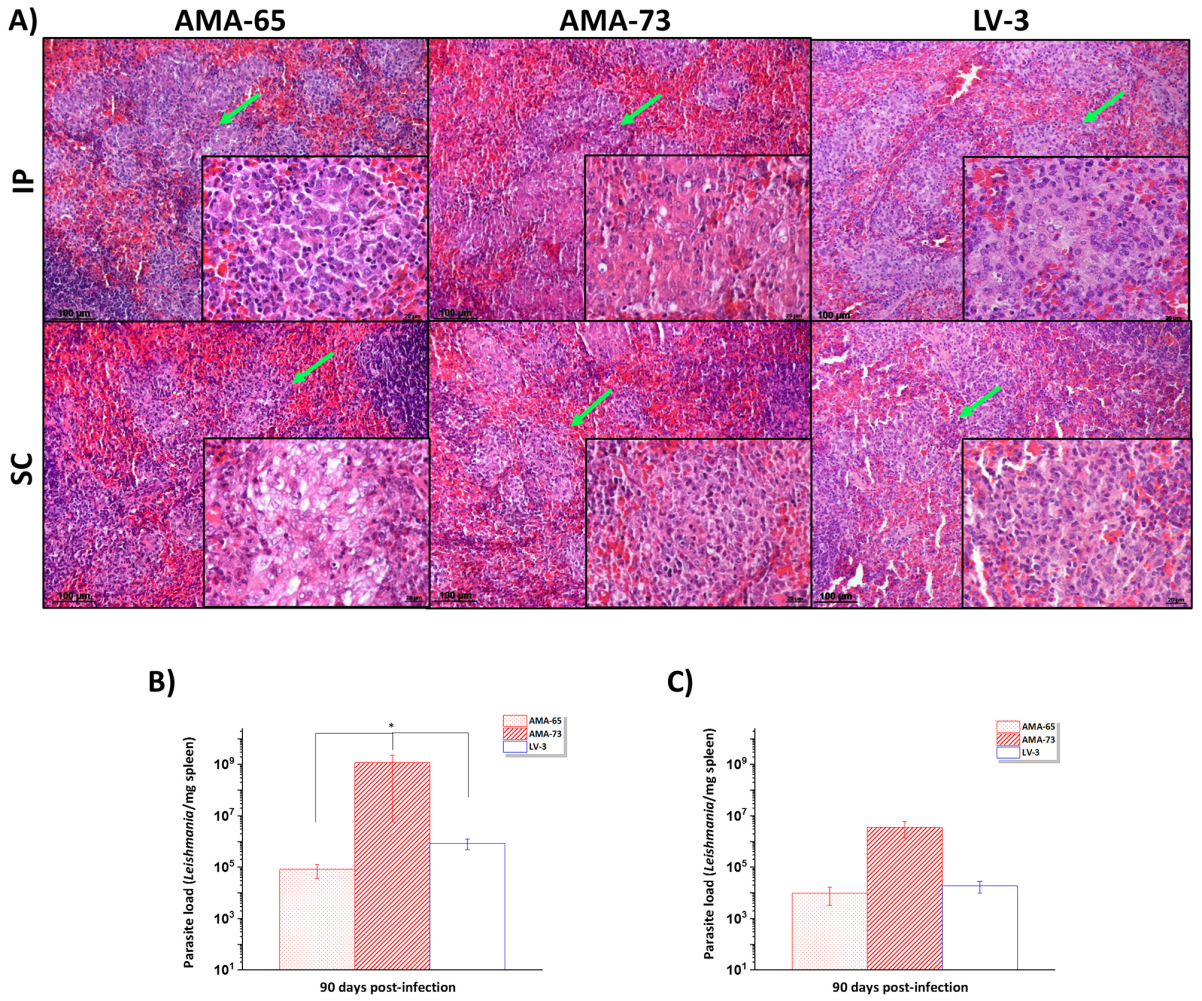
Histopathological changes and parasite load (parasites/mg) in the spleen after intraperitoneal (IP) and subcutaneous (SC) infection with AMA-65 and AMA-73 (dermotropic) and LV-3 (viscerotropic) *L.* (*L.*) *infantum chagasi* strains: (A) Histological sections of the spleen showing macrophages nodules in the splenic red pulp (green arrows). The type of inflammatory cells are highlighted. Parasite load in the spleen of hamsters infected by (B) the intraperitoneal route and (C) the subcutaneous route. * p < 0.05.

Our previous studies have suggested that a heightened pro-inflammatory response in the skin may hinder vascularization by dermotropic Honduran strains^17,18^. Based on this finding, our next step was to inject those parasites directly (IP and SC) into hamsters to bypass this barrier and facilitate their access to the viscera. After 90 days post-infection, both inoculation routes successfully established the parasites in the spleen. No substantial histopathological differences were detected among the dermotropic (AMA-65 and AMA-73) and viscerotropic (LV-3) strains, regardless of the inoculation route (Figure 4A). Overall, in the spleen, the white pulp remained preserved; however, hyperplasia and hypertrophy of macrophages in the red pulp led to nodule formation.

The inoculation route (SC versus IP) showed no impact on parasite establishment or histopathological features (Figure 4A). However, it did influence parasite load when inoculation was performed via the IP route. The dermotropic AMA-73 strain exhibited the highest tissue parasitism in the spleen (*p* < 0.05) (Figure 4B). After SC inoculation, the parasite load was less pronounced than that following IP inoculation, with no significant differences among the strains in the spleen (Figure 4C) (*p* > 0.05).

## DISCUSSION

*L.* (*L.*) *infantum chagasi* is a member of the *L.*(*L.*)*donovani* complex, often regarded as a causative agent of VL. However, atypical skin lesions caused by this species have been reported in individuals with no prior history of VL in both the Old and New Worlds. Clinical presentations vary by geographic region. For instance, in the Old World^19,20^ and some South American countries^21,22^, cutaneous lesions are typically ulcerative, whereas in Central America they are not, regardless of evolution time^4,23^. In this study, we analyzed the *in vitro* and *in vivo* biological behavior of four strains isolated from patients with NUCL, with a mean age of 38 years (ranging from 4–69 years), and one strain from a 1-year-old patient diagnosed with VL. All patients were from endemic regions where both clinical conditions are observed; however, it is noteworthy that NUCL and VL never occur concurrently in the same individual, nor does one condition precede the other^3,4^. Various factors may contribute to this divergence in clinical expression of the disease caused by the same parasite species. A plausible factor is the genetic and immunological background of the host. Thus, a possible explanation for the development of VL in the 1-year-old patient could be related to the status of the immune system, which is still maturing and may render the patient more susceptible to severe forms of the disease^24^. Moreover, data from the Ministry of Health of Honduras indicate that cases of VL predominantly impact children under the age of 5, with the highest incidence noted in those under 2 years old, whereas cases of NUCL are primarily reported in children over 5 and in adults^11^.

The limited reports in the literature regarding the *in vitro* and *in vivo* behavior of *L*. (*L.*) *infantum chagasi* strains causing NUCL and VL in Honduras^3^ encouraged us to evaluate their infectivity in peritoneal macrophages and hamsters. The hamster was chosen as an experimental model due to its susceptibility to infection by viscerotropic strains, such as *L. infantum* ^25^ and *L. donovani* ^26^. All strains employed in this study were characterized and exhibited the same profile, regardless of their dermotropic or viscerotropic profile as *L.* (*L.*) *infantum chagasi*. This characteristic confirmed their taxonomic status before the *in vitro* and *in vivo* procedures. Although they belong to the same species, detailed biological comparisons of these strains in animal models remain scarce^9^.

Since temperature variations occur in various host compartments, our initial step was to evaluate whether such variations would affect the infection index (II) in hamster macrophages by dermotropic and viscerotropic *Leishmania* strains. Intraspecies studies using different temperatures may explain why growth patterns influence parasite development under *in vitro* conditions. Previous *in vitro* studies involving peritoneal macrophages from BALB/c mice infected with Mediterranean (VL and CL) and Honduran (VL and NUCL) *L*. (*L.*) *infantum* strains revealed differences in infectivity. Mediterranean *L.* (*L.*) *infantum* strains were more infective than Honduran strains at 37 °C, whereas Honduran strains exhibited similar II between VL and NUCL^3^. When using the hamster model, an intriguing trend was observed *in vitro*: dermotropic strains showed a higher II at lower temperatures (32 and 34 °C), whereas the viscerotropic strain preferred higher temperatures (34 and 36 °C) during extended periods (48 h post-infection). In this study, we used macrophages from hamsters, an experimental model highly susceptible to *L*. (*L*.) *infantum* infection compared to BALB/c mice, which show a self-limited disease^26^. In addition, studies have shown that differences in parasite-host cell interaction are common, with a varying capacities for infection between different strains of the same species^27^. These data suggest that the strain (viscerotropic x dermotropic) may influence its development favorably at high or low temperatures in distinct host compartments. However, after internalization by macrophages, a critical step for dermotropic strains is to understand how they impact the production of inflammatory mediators compared to viscerotropic strains.

The survival of *Leishmania* within macrophages relies on its capacity to evade the microbicidal mechanisms exhibited by these cells^28^. Macrophage activation mechanisms are characterized by a cascade of reactions that trigger the production of reactive oxygen intermediates [hydrogen peroxide (H^2^O^2^) and nitric oxide (NO)] and cytokines to activate the immune response and facilitate parasite killing^29^. High concentrations of H^2^O^2^ compromised *L.* (*L.) donovani* motility and increased parasite lysis, suggesting that exposure to oxidative stress impedes its survival^30^. Moreover, it has been demonstrated that H^2^O^2^ can eliminate both promastigotes and amastigotes of *L.* (*L.) donovani*, although amastigote forms are slightly more resistant^31^. Using resident peritoneal macrophages, we could not detect H^2^O^2^ since its concentrations were below the detection threshold of the kit. This result may be due to the fact that our macrophages were not IFN-γ-primed, a condition that favors cell activation^32^. Another possibility is that *Leishmania* parasites possess a set of antioxidant enzymes that mitigate oxidant molecules, such as H^2^O^2 33^. The production of such parasitic enzymes may represent virulence factors, ensuring the parasite's maintenance within the vertebrate host^34^.

Subsequently, we aimed to investigate another inflammatory mediator, nitric oxide (NO). An association between a higher II and lower NO production by macrophages infected with different strains of *Leishmania* has been reported^27,35^. Moreover, it has been shown that LPG from dermotropic strains of *L.* (*L.*) *infantum chagasi* isolated from NUCL patients induces higher levels of NO than LPG from viscerotropic strains in IFN-γ-primed macrophages of BALB/c and C57BL/6 mice^36^. However, in the present study, no correlation between NO and II was found in hamster peritoneal macrophages infected with various strains of *L. (L.) infantum chagasi* isolated from NUCL and VL patients. Nonetheless, *in vitro* studies suggest that murine macrophages stimulated with IFN-γ and/or LPS and infected with *Leishmania* result in a marked increase in NO production^29^ which does not reflect the conditions of the present experiment and could explain the discrepancies in the results. Additionally, hamsters produce low amounts of NO, which may contribute to their susceptibility to infection. Studies have reported that hamsters infected with viscerotropic parasite strains, such as *L.* (*L.) donovani*, may exhibit a higher expression of pro-inflammatory cytokines, such as IFN-γ and IL-2. However, these animals demonstrate a failure in NO production via NOS2, which is an essential factor for controlling intracellular microorganisms^26^. Consistent with these observations, it is evident that *L.*(*L.*)*infantum chagasi* does not elicit a very strong immune response from resident hamster macrophages, which likely contributes to the high susceptibility of this model to this species both *in vitro* and *in vivo*.

It is important to emphasize that *in vitro* studies represent only a fraction of the overall picture, as they do not fully replicate the complexities of *in vivo* infection, in which other immune components play a role in shaping the host's innate and adaptive immune response, impacting disease outcome^37^. Our previous studies^17^ showed that the significant immune response observed in patients led us to hypothesize that the skin could serve as a strong barrier for the parasite, preventing its vascularization. This is completely different from viscerotropic *L.* (*L.*) *donovani*, which can visceralize using IL-1β and exosomes as crucial factors for cell attraction and systemic circulation^38^. On the other hand, recent *in vivo* studies using the hamster as an experimental model, with intraperitoneal and subcutaneous inoculation, demonstrated that isolates from patients with NUCL could reach the liver and spleen, causing visceral lesions similar to those induced by parasites isolated from human VL^9^. This *in vivo* result confirms that hamsters are a suitable model for experimental infection with dermotropic and viscerotropic Honduran strains, suggesting that the immune response established by the vertebrate host is critical in determining the phenotype of the infection caused by *L*. (*L*.) *infantum chagasi* in Central America. Thus, as indicated in our previous study^9^, our next step was circumventing this barrier by inoculating *L. (L.) infantum chagasi* strains via two different routes (IP and SC). After 90 days post-infection, hamsters infected with dermotropic (AMA-65 and AMA-73) and viscerotropic (LV-3) strains of *L. (L.) infantum chagasi* exhibited similar parasite loads and histopathological changes in the spleen, irrespective of the administration route. This finding likely relates to the chronicity of infection and intense tissue parasitism. Dermotropic strains could reach the spleen and induce histopathological changes akin to those caused by the viscerotropic strain. This result reinforces previous observations^9^ that hamsters are a susceptible model for experimental infection with both dermotropic and viscerotropic Honduran strains. Notably, intraspecies variations in parasite load were detected based on the administration route (IP x SC). Intraperitoneal injection proved more effective in inducing higher parasite loads in the assessed organs. Overall, the dermotropic AMA-73 strain exhibited a higher parasite load in the viscera via the IP route.

Despite minimal divergence, intraspecies variation among *L. infantum* isolates from human and canine cases has been reported, with no correlation observed between intraspecies divergence and the geographic distribution of the isolates^39^; a multi-kilobase deletion was recently detected in the genomes of some *L. infantum* strains, which was associated with reduced susceptibility to the anti-leishmanial drug^40^. If these intraspecies variations are present in samples isolated from Southern Honduras, they could explain the conflicting results, serving as one of the factors responsible for the differences in the biological behavior of these samples. As previously noted, the data presented by Flores *et al*.^9^ reinforce the notion that when allowed to reach the viscera, dermotropic *L. (L.) infantum chagasi* strains from Honduras may induce histopathological changes similar to those observed in the viscerotropic strain from Honduras and the Brazilian reference strain (MHOM/BR/1972/BH-46)^7^. One of the questions warranting further investigation is the role of vector-derived factors in *L. evansi* and *L. longipalpis* in NUCL endemic areas of Honduras, which may influence their migration toward organs and favor their remaining in the skin. This underscores the complexity of host-parasite interactions in *Leishmania,* which rely on multiple components working in combination, including the parasite, the host, and the vector.

## CONCLUSION

Honduran dermotropic (NUCL) and viscerotropic (VL) strains of *L. (L.) infantum chagasi* exhibited a growth cline in hamster macrophages under varying temperatures *in vitro*. These strains triggered a minimal immune response in macrophages, likely due to the low responsiveness of the hamster model and the immunosuppressive characteristics of *L. (L.) infantum chagasi*. The *in vivo* study demonstrated that both dermotropic and viscerotropic Honduran strains could induce VL in hamsters, confirming the suitability of this model for these strains. However, further research is necessary to deepen our understanding of the host-parasite relationship in *L*. (*L*.) *infantum chagasi* infections in Central America, which, in addition to manifesting as visceral disease, also causes the rare non-ulcerated cutaneous form of the disease.
